# The safety of blinatumomab in pediatric patients with acute lymphoblastic leukemia: A systematic review and meta-analysis

**DOI:** 10.3389/fped.2022.929122

**Published:** 2022-07-22

**Authors:** Maria Maddalena Marrapodi, Annamaria Mascolo, Gabriella di Mauro, Gianluca Mondillo, Elvira Pota, Francesca Rossi

**Affiliations:** ^1^Department of Woman, Child and General and Specialist Surgery, University of Campania “Luigi Vanvitelli”, Naples, Italy; ^2^Campania Regional Centre for Pharmacovigilance and Pharmacoepidemiology, Naples, Italy; ^3^Department of Experimental Medicine – Section of Pharmacology “L. Donatelli”, University of Campania “Luigi Vanvitelli”, Naples, Italy

**Keywords:** blinatumomab, B-cell acute lymphoblastic leukemia, safety, cytokine release syndrome, neurologic events, pediatric

## Abstract

**Background:**

Blinatumomab is a bispecific CD19-directed CD3 T-cell engager that has proven efficacy in children with relapsed or refractory B-cell acute lymphoblastic leukemia (ALL). Despite its efficacy, it has also been associated with the development of potentially serious adverse events such as the cytokine release syndrome (CRS) and neurologic events. The present meta-analysis aimed to assess the safety profile of blinatumomab in terms of serious adverse events, CRS, and neurologic events (such as seizure and encephalopathy) in pediatric patients with B-cell ALL.

**Methods and findings:**

A systematic review was conducted in Pubmed up to December 10, 2021 to retain pediatric clinical trials on blinatumomab. A random effect meta-analysis approach was used. This study followed the PRISMA statement. Four out of the 255 initial references were selected, of which 2 were phase 1/2 clinical trials and 2 phase 3 clinical trials. Blinatumomab was associated with a lower risk of serious adverse events (Risk ratio RR, 0.56; 95% CI, 0.32–0.99), febrile neutropenia (RR, 0.13; 95% CI, 0.06–0.26), infection (RR, 0.40; 95% CI, 0.29–0.56), and grade ≥ 3 adverse events (RR, 0.79; 95% CI, 0.67–0.93) compared to chemotherapy. No difference in the risk of CRS (RR, 8.37; 95% CI, 0.27–260.97) and seizure (RR, 6.43; 95% CI, 0.79–53.08) was observed between groups, while for encephalopathy a higher risk was associated with blinatumomab compared to chemotherapy (RR, 8.90; 95% CI, 1.08–73.29).

**Conclusion:**

Our data support the good safety profile of bliantumomab in treating pediatric patients with B-ALL.

## Introduction

Acute lymphoblastic leukemia (ALL) is a form of cancer very common in children, with an incidence of about three cases per 100,000 persons per year (1, 2). Relapse in patients are associated with a cure rate ranging from 10 to 70% that varies based on the site and time to relapse (2). Children with relapsed or refractory ALL have a bad prognosis, even if they are in treatment with intensive combination chemotherapy and allogeneic hematopoietic stem-cell transplantation (alloHSCT) (2, 3). Survival for these patients is poor, especially for those with early relapse (5-year survival ranged 25–50%) and the remission induction therapy is challenging due to cumulative chemotherapeutic toxicities (3–7). However, in recent decades, emerging immunotherapies constitute an important new antileukemic treatment strategy, showing promising results in terms of circumventing the problems of chemo-resistance and previous toxic organ damage (3). Among them, blinatumomab, a bispecific T cell–engaging monoclonal anti-body that links CD3 + T cells to CD19 + leukemia cells and induces an immune response of cytotoxic T lymphocytes (8), has shown the ability of causing a lysis of target cell *in vitro* and an antitumor activity *in vivo* (9). Moreover, blinatumomab has demonstrated an antileukemic activity in clinical studies conducted in children with relapsed or refractory B-cell ALL (2) and high rates of complete response in patients with molecularly resistant B-cell ALL (10). Based on the encouraging data, blinatumomab was approved by the US Food and Drug Administration for the treatment of “pediatric patients with B-cell precursor acute lymphoblastic leukemia (ALL) who are in remission but still have minimal residual disease (MRD)” and by the European Medicine Agency for the treatment of “pediatric patients aged 1 year or older with Philadelphia chromosome negative CD19 positive B-precursor ALL, which is refractory or in relapse after receiving at least two prior therapies or in relapse after receiving prior allogeneic haematopoietic stem cell transplantation, and as monotherapy for the treatment of pediatric patients aged 1 year or older with high-risk first relapsed Philadelphia chromosome negative CD19 positive B-precursor ALL as part of the consolidation therapy” (11, 12). Despite its efficacy and based on its mechanism of action, blinatumomab was also associated with the development of potentially life-threatening conditions related to the non-physiologic activation of T cells such as the cytokine release syndrome (CRS) (13). Moreover, clinical studies found that blinatumomab is also associated with the development of neurologic events, which are considered the most frequent reason for drug discontinuation (14). However, no meta-analysis of pediatric clinical trials has been conducted to evaluate the safety profile of blinatumomab. Therefore, considering this and the seriousness of such events, we decided to perform a meta-analysis of pediatric clinical trials to evaluate the safety profile of blinatumomab in terms of serious adverse events, cytokine release syndrome (CRS), neurologic events, and events potentially associated with life-threatening complications (such as infection and febrile neutropenia) in pediatric patients with B-ALL.

## Methods

### Search strategy and selection criteria

The Preferred Reporting Items for Systematic Reviews and Meta-Analysis (PRISMA) guideline were used to perform a standardized data search, extraction, reporting, and presentation. A systematic literature search of clinical trials of blinatumomab in pediatric patients with ALL published up to December 10, 2021 was performed in PubMed. The search strategy is shown in supplementary file 1 and it was performed by two investigators (AM and MM) who screened the scientific literature. One investigator identified selection criteria and started the review of titles and abstracts of all retrieved studies (MM). Another investigator validated these criteria and independently screened the selected papers (AM). In case of discrepancies between the two investigators, a third investigator was consulted (FR) to get a final decision. A population/participant, intervention, comparator/control, outcome, study design, known as PICOS, framework was developed. Population: pediatric patients with acute lymphoblastic leukemia; Intervention: blinatumomab; Comparator: standard chemotherapy; Outcome: number of adverse events, serious adverse events, adverse events graded ≥ 3, cytokine release syndrome (CRS), encephalopathy, seizure, neurologic events, infection, or febrile neutropenia; Study design: clinical trial. We included articles that met the PICOS criteria. Articles in languages other than English were excluded. Reviews, letters, commentary, case reports/series, editorials, preclinical studies, guidelines, observational studies, or other type of studies (including studies on genetics, pharmacoeconomic, *post-hoc* analysis, etc.) were also excluded.

### Data extraction

The following data were extracted from each selected article,: year of publication, number of patients, number of patients participating to the safety analysis, number of male and female, phase of the clinical trial, age, dose of blinatumomab, bone marrow blast count, and type and number of outcome events. For phase 3 clinical trials the information was reported for each arm (blinatumomab vs. comparator).

### Endpoint and statistical analysis

For phase 3 clinical trials, the endpoint was to assess the risk ratio of adverse events, serious adverse events, adverse events graded ≥ 3, cytokine release syndrome (CRS), encephalopathy, seizure, and neurologic events for blinatumomab compared with comparator arm. A random effect meta-analysis was applied, with the Q^2^ test statistics used to evaluate the heterogeneity of the effect across studies. A Q statistic with a *p*-value < 0.10 was considered significant. The percentage of total variation across studies due to heterogeneity rather than chance was assessed with I^2^ statistics, which values <25% were used for low heterogeneity, between 25 and 75% for moderate heterogeneity, or > 75% for high heterogeneity. Risk ratios (RRs) and 95% confidence intervals (95% CIs) were computed for dichotomous outcomes. Results were also displayed as fixed effect model. For phase 1/2 and 3 clinical trials data were pooled to estimate the incidence rate of CRS and neurologic events. Incidence rates were computed by dividing the number of events with the number of pediatric patients exposed.

Statistical analyses were computed with software R version 3.6 (R Foundation for Statistical Computing, Vienna, Austria).

## Results

### Study selection and characteristics

**Figure 1:**
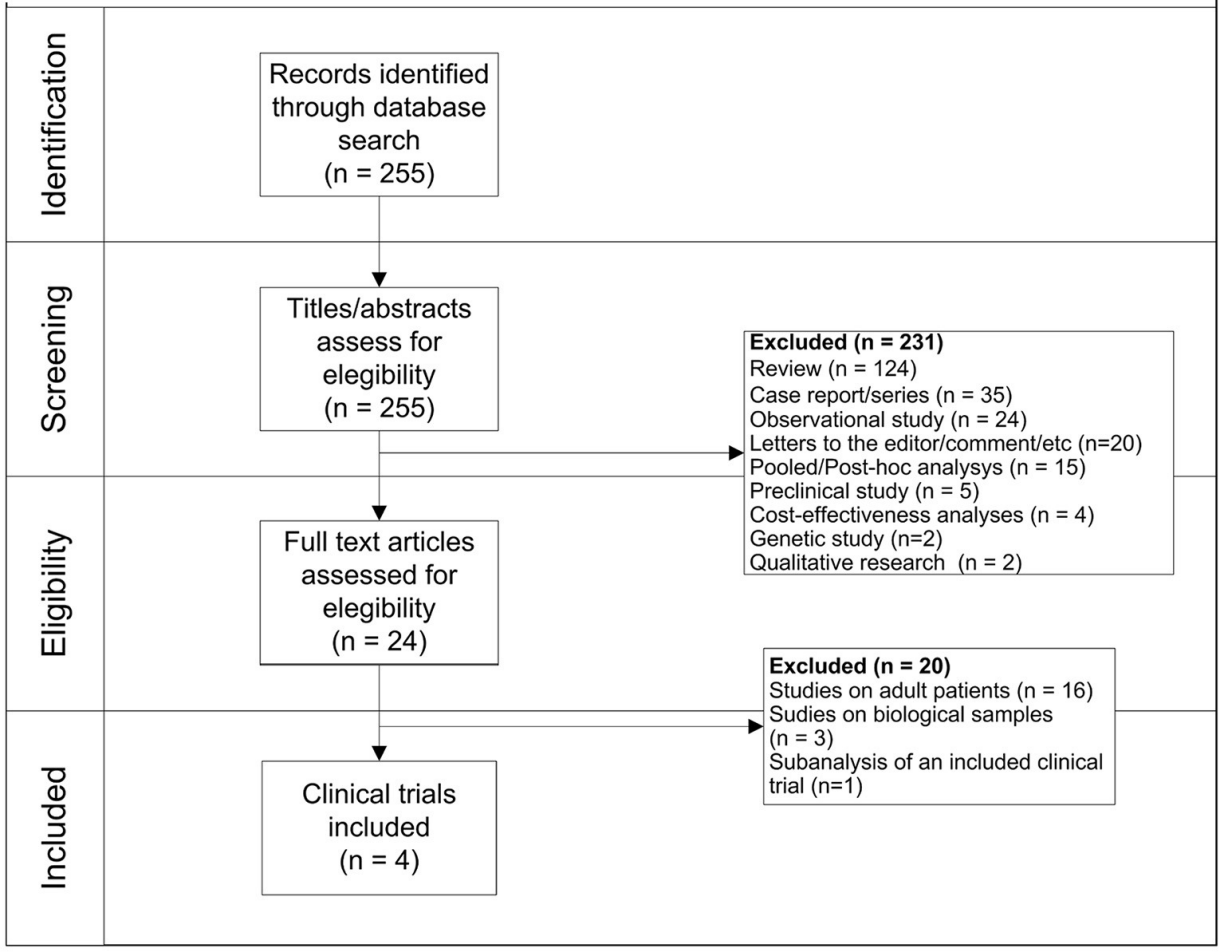
Flow-chart for the identification of eligible clinical trials.

Two hundred fifty-five records were firstly identified, of which 231 were initially excluded because they were not clinical trials. Specifically, we excluded records because they were: review (*N* = 124), case report/series (*N* = 35), observational studies (*N* = 24), letters/commentary (*N* = 20), *post-hoc* analysis (*N* = 15), preclinical studies (*N* = 5), cost-effectiveness analyses (*N* = 4), genetic studies (*N* = 2), and qualitative researches (*N* = 2). Observational studies were primarily excluded because they do not fall within our aim of assessing the safety profile of blinatumomab by using pediatric clinical trials. Moreover, because they investigated other therapies rather than blinatumomab (*N* = 8), they were conducted in adults (*N* = 5), they were not in English (*N* = 1), or they were small studies without a comparison group (*N* = 6). Then, a total of 24 clinical trials was identified, of which 20 were excluded because they were conducted in adult patients (*N* = 16), they were related to biological samples (*N* = 3) or were sub-analyses of already included clinical trials (*N* = 1). Finally, 3 (19%) of aforementioned clinical trials conducted in adult patients evaluated the efficacy of chimeric antigen receptor (CAR) T-cell therapy rather than blinatumomab. As a result, a total of 4 clinical trials was identified, of which 2 were phase 1/2 clinical trials (2, 15) and 2 were phase 3 clinical trials (16, 17) (Figure 1). General characteristics of clinical trials are showed in Table 1.

**Table 1:** Characteristics of pediatric clinical trials on blinatumomab.

**Author**	**Year of publ ication**	**Number of patient enrolled**	**Blin atumomab arm (** ***N*** **)**	**Compa rator arm (** ***N*** **)**	**Male/ Female blin atumomab (** ***N*** **)**	**Male/ Female comparator (** ***N*** **)**	**Phase**	**Age blin atumomab arm median (range)**	**Age comparator arm median (range)**	**Bone marrow blast count**	**Blin atumomab dosage**	**Outcome included**
von Stackelberg A et al. (2)	2016	70	70	-	47/23	-	Phase 1/2	8 (1–17)	-	25%	5 μg/m2/d x 1 w 15 μg/m2/d x 3 w^**^	- CRS - Neurologic events
Horibe K et al. (15)	2020	10	9	-	4/5	-	Phase 1	11 (7–17)	-	5%	5 μg/m2/d x 1 w 15 μg/m2/d x 3 w ^**^	- CRS - Neurologic events
Locatelli F et al. (16)	2021	108	54	54	30/24	22/32	Phase 3	6 (1–17)	5 (1-17)	5% but <25%	15 μg/m2/d x 4 w	- Number of adverse events - Serious adverse events - Grade ≥3 adverse events - CRS - Encephalopathy - Seizure
Brown PA et al. (17)	2021	208	105	103	57/48	54/49	Phase 3	9 (6–16)	9 (5–16)	-	15 μg/m2/d^#^	- Number of adverse events - Serious adverse events - Grade ≥3 adverse events - CRS - Encephalopathy - Seizure

*Cytokine Release Syndrome (CRS) ^**^Patients received blinatumomab as a 4-week continuous intravenous infusion, followed by a 2-week treatment-free interval. The lower dose was administered for the first week of the first cycle followed by the higher dose for the remaining 3 weeks and subsequent cycles. # 2 continuous 28-day infusion cycles of blinatumomab at 15 μg/m2 per day, separated by a 1 week break.

### Phase 1/2 clinical trials

Two phase 1/2 clinical trials were identified involving a total of 79 patients with B-cell acute lymphoblastic leukemia (B-ALL). Among safety outcomes both studies evaluated neurologic events and CRS. One open-label clinical trial enrolled 9 Japanese children with B-ALL, who received blinatumomab for 4 weeks (at a dose of 5 μg/m2/day for week 1 and followed by a dose of 15 μg/m2/day for weeks 2–4), followed by a treatment-free interval of 2-week. In this study a total of 7 neurologic events and 5 CRS were identified (15). The other open-label trial enrolled children aged < 18 years with relapsed/refractory (B-ALL), with a total of 70 patients that received the recommended dosage using 6-weeks treatment cycles. During the study, a total of 17 neurologic events and 8 CRS were observed (2). The estimated pooled incidence rate was 0.21 (95% CIs 0.16–0.27) for neurologic events and 0.16 (95% CIs 0.11–0.21) for CRS (Table 2).

**Table 2:** Incidence Rate of Cytokine Release Syndrome and neurologic events of pooled phase 1/2 and 3 clinical trials.

**Author**	**Number of events**	**Total exposed**	**Incidence rate (95% Confidence Interval)**
**Cytokine Release Syndrome**	37	235	0.16 (0.11–0.21)
**Neurologic events**	50	235	0.21 (0.16–0.27)

### Phase 3 clinical trials

One randomized phase 3 clinical trial included children aged > 28 days and < 18 years with B-ALL at high-risk of first-relapse with morphologic complete remission (M1 marrow, <5% blasts) or M2 marrow (blasts ≥ 5 and <25%) at time of randomization. These patients were previously treated with induction therapy and 2 blocks of consolidation therapy and then randomized to receive a third consolidation course with 1 cycle of continuous intravenous infusion of blinatumomab for 4 weeks (dose: 15 μg/m2/day) or chemotherapy (16).

**Figure 2:**
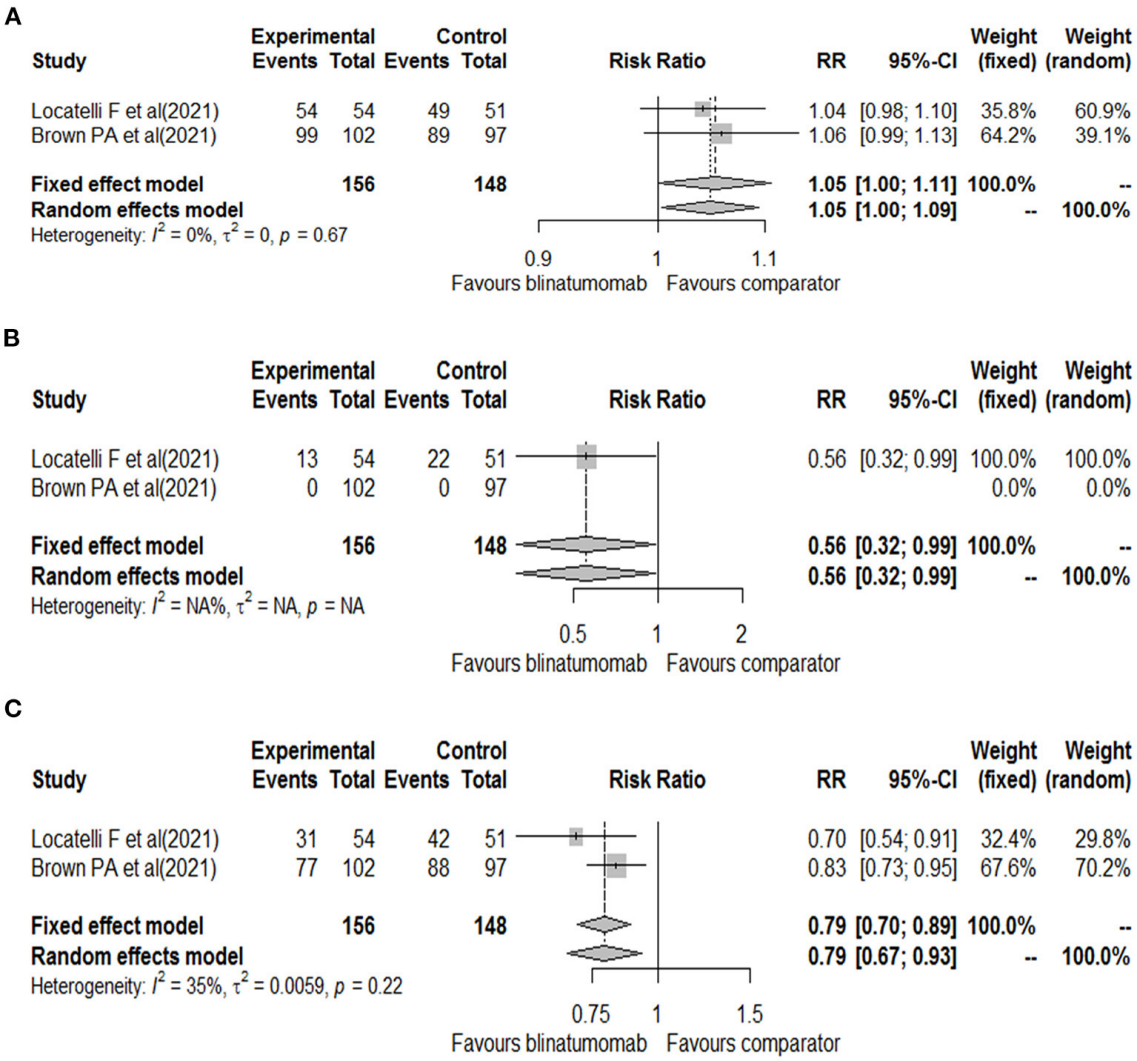
Forest plot of total adverse events **(A)**, serious adverse events **(B)**, and grade ≥ 3 adverse events **(C)** with blinatumomab vs. comparator.

**Figure 3:**
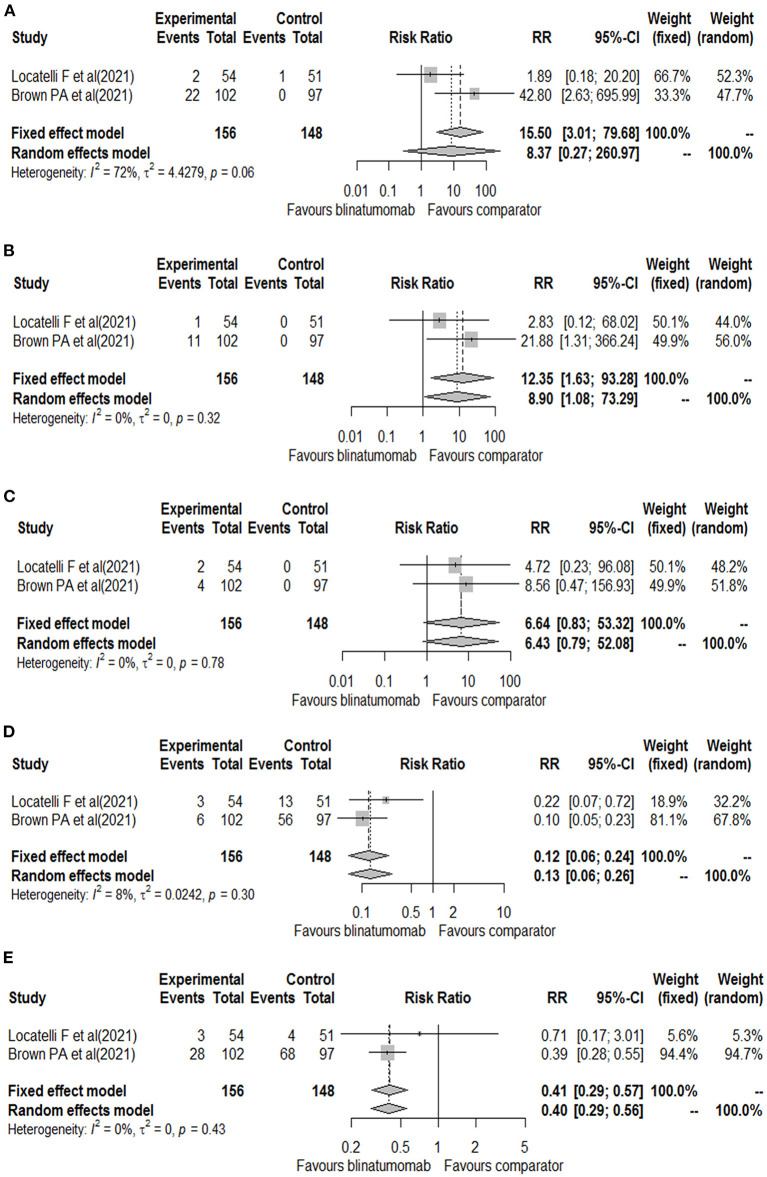
Forest plot of cytokine release syndrome **(A)**, encephalopathy **(B)**, seizure **(C)**, febrile neutropenia **(D)**, and infections **(E)** with blinatumomab vs. comparator.

The other randomized phase 3 clinical trial included patients aged 1 to 30 years with B-ALL at high- and intermediate-risk of first relapse. The trials enrolled a total of 208 patients, of which 33 (15.9%) were aged 18–30 years (15 of blinatumomab group and 18 of comparator group). All patients were treated with a course of reinduction chemotherapy for 4-weeks, followed by 2 cycles of blinatumomab or multiagent chemotherapy based on randomization (17). Based on the different treatment schedules, data on the safety outcomes were retrieved after the first cycle of blinatumomab for both clinical trials (16, 17). These two clinical trials evaluated all our safety outcomes with the exception of the total number of neurologic events that was reported for only one study (26 for blinatumomab vs. 15 for comparator) (16). Therefore, all other safety outcomes were meta-analyzed. For the total number of adverse events, 153 adverse events were found in the blinatumomab group and 138 in the comparator group. No difference in the risk of total adverse events was observed between blinatumomab and chemotherapy (RR, 1.05; 95% CI, 1.00–1.09; Figure 2A), with consistency across studies (I2 = 0%; *p* = 0.67). For the number of serious adverse events, 13 events for blinatumomab and 22 for chemotherapy were observed. Blinatumomab was associated with a lower risk of serious adverse events compared to chemotherapy (RR, 0.56; 95% CI, 0.32–0.99; Figure 2B). For the number of adverse events graded ≥ 3, 108 and 130 events were observed for blinatumomab and chemotherapy, respectively. A lower risk of grade ≥ 3 adverse events was found with blinatumomab compared to chemotherapy (RR, 0.79; 95% CI, 0.67–0.93; Figure 2C), with moderate heterogeneity (I2 = 35%; *p* = 0.22). For CRS, 24 events were found in the blinatumomab group and 1 event in the comparator group. No difference in the risk of CRS was observed between groups (RR, 8.37; 95% CI, 0.27–260.97; Figure 3A), with moderate heterogeneity (I2 = 72%; *p* = 0.06). For encephalopathy a total of 12 events were observed with blinatumomab, while no event was observed with the control group. Blinatumomab showed a higher risk of encephalopathy compared to chemotherapy (RR, 8.90; 95% CI, 1.08–73.29; Figure 3B), with no observed heterogeneity (I2 = 0%; *p* = 0.32). Finally, 6 events of seizure were observed only in the blinatumomab group. No difference in the risk of seizure was observed between the two groups (RR, 6.43; 95% CI, 0.79–53.08; Figure 3C), with no observed heterogeneity (I2 = 0%; *p* = 0.78). Among the events potentially associated with life-threatening complications, 9 events of febrile neutropenia occurred with blinatumomab and 69 with the comparator group. Blinatumomab showed a lower risk of febrile neutropenia then the comparator arm (RR, 0.13; 95% CI, 0.06–0.26; Figure 3D), with a low heterogeneity (I2 = 8%; *p* = 0.30). The number of infection were 31 for the blinatumomab group and 72 for the comparator group, with a lower risk for blinatumomab than the comparison (RR, 0.40; 95% CI, 0.29–0.56; Figure 3E) and consistency across studies (I2 = 0%; *p* = 0.43).

## Discussion

The present meta-analysis is the first to our knowledge that evaluated the safety profile of blinatumomab with a focus on the risk of encephalopathy, seizure, and CRS in the pediatric population. A previous meta-analysis on blinatumomab included both adult and pediatric patients and reported data published up to 2017, thus not including pediatric phase 3 clinical trials (12). Therefore, an important strength is that we considered only pediatric clinical trials in spite of the limited number retrieved (4 eligible clinical trials), which highlights the need of further researches in this field (18).

We observed an incidence rate of 0.21 (95% CIs 0.16–0.27) for neurologic events and 0.16 (95% CIs 0.11–0.21) for CRS. The previous meta-analysis found instead a lower occurrence rate of CRS (0.04; 95%CIs 0.01–0.06) and neurologic events (0.12; 95% CIs 0.08–0.16), but we should consider that these rates were estimated only for grade 3 events and by considering a mixed population (adult and pediatric) (12). In our meta-analysis, blinatumomab showed a good safety profile with a lower risk of serious adverse events, grade ≥ 3 adverse events, and events potentially associated with life-threatening complications compared to chemotherapy, while no difference was found for the total number of adverse events. These results are consistent with those expected in patients with limited leukemia burden and are important for considering blinatumomab as an alternative therapy to standard consolidation chemotherapy. Indeed, standard consolidation chemotherapy after induction chemotherapy is commonly associated with the development of serious events that may result in death or reduce the likelihood of proceeding to alloHSCT (19). For the risk of CRS, blinatumomab showed no difference compared to chemotherapy. This could be attributed to the low number of cytokine release syndrome events that could be likely related to the low leukemic burden of patients, especially for the trial of Locatelli et al. (16), thus confirming what is supported by studies conducted on the adult population (2, 20, 21). Indeed, CRS is a systemic inflammatory response triggered by the activation of effector T cells upon their interaction with targets (13, 19). The clinical feature of this syndrome is the presence of high levels of inflammatory cytokines and biomarkers, such as the interleukin-6 (IL-6), ferritin, interferon-?, and C-reactive protein that substantially exceeds physiological levels. The first clinical sign of CRS is fever followed by malaise, but CRS can also evolve to capillary leak syndrome and vasodilatory shock with hypoxia and pulmonary failure (13). The onset and severity of CRS can depend by the presence of risk factors such as disease burden and initial starting dose of blinatumomab (13, 22–25). In general, signs and symptoms developed during the first cycle of blinatumomab (13). According to this and our results, a study showed that grade 3 CRS occurred only in 2% of patients, whereas the 60% developed only pyrexia. In this study, it was performed a prophylaxis for CRS with the administration of dexamethasone on day 1 and dose escalation, and a cytoreduction for those patients at higher disease burdens (26). The prophylaxis with dexamethasone was also executed in one of the included clinical trial (16), highlighting the importance of prophylaxis regimen in preventing first-dose adverse events. Currently, risk mitigation strategies for blinatumomab-induced CRS are a strategy at step-dose during the first cycle of treatment, the use of prophylactic regimen with dexamethasone before starting the infusion, and a close monitoring of patients during the period at high risk of CRS (27). However, other prophylactic strategies are being investigated. Tocilizumab, a monoclonal antibody that inhibits the receptor of IL-6, was approved for the treatment of severe or life-threatening CRS related to chimeric antigen receptor T (CAR-T) cell (27). Evidence also showed the potential of using prophylactic tocilizumab to reduce incidence and severity of CRS (28). Moreover, it should be preferred to dexamethasone because it does not affect the efficacy of blinatumomab, while dexamethasone can inhibit T cell proliferation and antitumor activity (29). Despite data on the prophylactic use of tocilizumab are cited in the Society for Immunotherapy of Cancer (SITC) clinical practice guideline on immune effector cell-related adverse events, they are not yet included among recommendations (30).

For the risk of neurologic events, blinatumomab showed a higher risk of encephalopathy compared to chemotherapy, while no difference was observed for the risk of seizure. This is in accordance with studies that reported among the most frequent neurologic events with blinatumomab: tremor, dizziness, confusional state, and encephalopathy (8, 26, 31–33). Among patients experiencing neurologic events, the median time to event was generally within the first 2 weeks of treatment, but it should be considered that encephalopathy can also develop during the second cycle of treatment, even in patients who did not suffer from this complication during the first administration (34). Among risk factors that can influence neurologic events, there are disease- or treatment-related factors, such as the use of two or more prior rescue therapies, and the prior history of neurologic disorders (14). Prevention strategies for neurotoxicity have also been considered in the literature. Evidence shown a reduction of neurotoxicity in patients under the antiepileptic treatment with levetiracetam (3). Moreover, intrathecal chemotherapy prior to infusion with blinatumomab might also be considered to reduce the risk of neurotoxicity (35). Intrathecal chemotherapy with hydrocortisone and cytotoxic agents to reduce inflammation and to eliminate the immune effector cells, respectively, was also found effective for the management of steroid-refractory neurotoxicity induced by CAR-T cells (36). Moreover, intrathecal chemotherapy could be an ideal strategy to reduce the use of systemic steroids on CAR-T cell activity, which is necessary for the control of disease (36). On the contrary of CRS, the disease burden and the blinatumomab dose does not influence the occurrence of such events (10, 14). Moreover, the mechanism underlying the development of neurologic events is not clear but likely associated with multiple factors (10). In this regard, since encephalopathy and seizure events, including severe and fatal ones, have also been reported in patients receiving CD19-targeted CAR-T cells (24, 37), there is the possibility of a target dependent mechanism (14). Moreover, evidence also suggested that a transient neuroinflammatory irritation may be the cause of blinatumomab-associated events (38), although the detailed pathogenesis is not completely elucidated.

Although blinatumomab has shown a significant survival benefit compared to chemotherapy, it has a particular toxicity profile that requires a scrupulous surveillance by all healthcare professionals (39), both during drug preparation and, above all, in the early stages of drug infusion. In fact, the preparation should be performed under aseptic conditions by qualified personnel. It is very important to strictly follow the preparation and administration instructions to minimize medication errors, including underdose, and overdose. From a microbiological point of view, the prepared infusion bags should be used immediately. Otherwise, the storage times during use and the conditions before use should not exceed 24 h at a temperature of 2°C−8°C, unless dilution has been performed under controlled aseptic conditions. Close monitoring during the first 72 h of treatment will be indicated because of the potential cytokine release effects triggered by the administration of blinatumomab. Nurses/physicians trained in emergency medicine should be available for immediate intervention in case of complications. The patient's organ functions should be closely monitored during blinatumomab administration and particular attention should be paid to the subject's mental status and neurologic function. An important role in limiting the severity of events is given by appropriate patient management, which must consider the patient history and the use of the aforementioned prophylactic therapies. All these recommendations for clinical surveillance highlight the importance of education and training of healthcare professionals.

### Limitations

The use of a single repository such as PubMed is an important limitation in our meta-analysis. Indeed, the meta-analysis was performed on a limited number of clinical trials, which also have differences in the characteristics of population and treatment schedules. Furthermore, in the trial by Brown et al. was not possible to select only events that occurred in the pediatric cohort, because it included patients aged up to 30 years. All this could have affected our results. Finally, conclusions could be susceptible to study's limitations, publication bias and the formulation of eligibility criteria. These limitations may create bias and alter the analyses. Therefore, results should be interpreted accordingly.

## Conclusions

In conclusion, blinatumomab showed a good safety profile in treating pediatric patients with B-ALL. The risk of serious adverse event was indeed higher with chemotherapy than blinatumomab. The occurrence of CRS and neurologic events was very low among clinical trials. Moreover, CRS and neurologic events have showed a generally good response to drug withdrawal and interventional therapies in the analyzed clinical trials. Moreover, an important role in limiting the seriousness of these events is recovered by the appropriate management of patients that has to consider the patients' history and the use of prophylaxis therapies. Limited progress in treating relapsed disease have been done in the last 20 years, with few new drugs that have been developed for ALL. CAR T cells demonstrated significant results in determining sustained and durable responses in patients not only with bone marrow relapse but also with relapse in the central nervous system. CAR T cells thus have the potential to be an alternative to HSCT as cure of relapsed disease in spite of the remaining concern represented by CRS, since complications of HSCT should not be underestimated as well. Current trials are investigating instead the use of inotuzumab ozogamicin in multidrug regimens, including a phase I study evaluating the safety of this drug in combination with prednisone, cyclophosphamide, and vincristine in patients affected by refractory CD22 + acute leukemia. However, further studies are needed to better evaluate blinatumomab safety profile and to allow the execution of more reliable meta-analytic approach.

## Data availability statement

The raw data supporting the conclusions of this article will be made available by the authors, without undue reservation. And I did not detect any particular expressions.

## Author contributions

AM, MM, and FR performed the screening of the literature. AM and MM performed the analysis and wrote the paper. AM, MM, GM, GM, EP, and FR drafted the work and revised it for important intellectual content. FR developed the concept and designed the study. All authors approved the final version of the manuscript. All authors contributed to the article and approved the submitted version.

## Conflict of interest

The authors declare that the research was conducted in the absence of any commercial or financial relationships that could be construed as a potential conflict of interest.

## Publisher's note

All claims expressed in this article are solely those of the authors and do not necessarily represent those of their affiliated organizations, or those of the publisher, the editors and the reviewers. Any product that may be evaluated in this article, or claim that may be made by its manufacturer, is not guaranteed or endorsed by the publisher.
