# Supplementary material for: The safety of blinatumomab in pediatric patients with acute lymphoblastic leukemia: A systematic review and meta-analysis
Source: Front Pediatr. 2022 Jul 22;10:929122. doi: 10.3389/fped.2022.929122 (PMC9354602; doi:10.3389/fped.2022.929122)
Supplement: Supplementary file 1 [file Table_1.docx]

1. **BLINATUMOMAB**

**Search algorithm**

**Search terms**: ((blinatumomab OR blincyto OR MT103 OR bispecific T-cell engager OR BiTE) AND (acute lymphoblastic leukemia OR ALL) AND (pediatric OR children OR child* OR adolescence OR adolescent))

**Publication date** to December 10, 2021
